# Statistical process control for data without inherent order

**DOI:** 10.1186/1472-6947-12-86

**Published:** 2012-08-06

**Authors:** Alan J Poots, Thomas Woodcock

**Affiliations:** 1 Imperial College, London and NIHR CLAHRC for NWL, Floor 4 Lift Bank D, Chelsea and Westminster Hospital, 369 Fulham Road, London, SW10 9NH, UK

**Keywords:** Statistical process control (SPC), Individual and moving range (XmR), Ordering of data

## Abstract

**Background:**

The XmR chart is a powerful analytical tool in statistical process control (SPC) for detecting special causes of variation in a measure of quality. In this analysis a statistic called the *average moving range* is used as a measure of dispersion of the data. This approach is correct for data with natural underlying order, such as time series data. There is however conflict in the literature over the appropriateness of the XmR chart to analyse data without an inherent ordering.

**Methods:**

We derive the maxima and minima for the average moving range in data without inherent ordering, and show how to calculate this for any data set. We permute a real world data set and calculate control limits based on these extrema.

**Results:**

In the real world data set, permuting the order of the data affected an absolute difference of 109 percent in the width of the control limits.

**Discussion:**

We prove quantitatively that XmR chart analysis is problematic for data without an inherent ordering, and using real-world data, demonstrate the problem this causes for calculating control limits. The resulting ambiguity in the analysis renders it unacceptable as an approach to making decisions based on data without inherent order.

**Conclusion:**

The XmR chart should only be used for data endowed with an inherent ordering, such as a time series. To detect special causes of variation in data without an inherent ordering we suggest that one of the many well-established approaches to outlier analysis should be adopted. Furthermore we recommend that in all SPC analyses authors should consistently report the type of control chart used, including the measure of variation used in calculating control limits.

## Background

Statistical process control (SPC) is an approach to quality improvement that has seen increasing use in healthcare since the early 1990s [1]. Originated by Shewhart [2], SPC provides analytical tools to understand the variation displayed by measures of quality, and an approach to taking action on the resulting information with a view to making improvements. The control charts that form the mainstay of SPC analysis provide a simple graphical approach to understanding variation. Following Shewhart’s initial work, SPC was subsequently developed by Shewhart and Deming [3], and substantial literature now exists, including on the application of SPC in healthcare [4-10].

The most natural application of SPC in healthcare is to time series data - the natural ordering of the data in time is central to the correct application of the analysis. However, in the SPC literature there are conflicting opinions on the usage of control charts for data that does not come endowed with a natural ordering. Some authors recommend the use of control charts for such data [4], and some have used this analysis, for example in comparing hazard ratios for specific mortality rates [8]. Other authors argue against the use of control charts in such situations [9]. In this article we resolve objectively and quantitatively the question of whether it is acceptable to use control charts to analyse variation in data that does not have a natural ordering.

As all measured data exhibit variation, the idea behind a control chart is to provide concrete rules to assess the likely nature of the observed variation. Broadly speaking, the observed variation is classified as either “common cause” variation or “special cause” variation. Common cause variation is the variation exhibited by a process in its usual state, whereas special cause variation is caused by an exceptional or external event. The rules are couched in terms of a number of horizontal lines - the process (or control) limits - marked on a line graph of the data. The calculations of these features depend on which type of chart one is using in the SPC setting, and there are several available. The most commonly used are the individual values and moving range (XmR) charts; p-charts (used to monitor the proportion of faults in a sample); np-charts (an adaptation of the p-chart used to interpret performance in numbers of units rather than proportion); c-charts (to monitor count data - number of faults per unit - or to monitor the total number of events occurring over certain unit of time); and u-charts (for monitoring count data with the sample size greater than one, i.e. the average number of faults per unit). The XmR chart is one of the simplest of the charts to construct, and yet also one of the more robust in general practice, as the other charts rely on the data conforming to an assumed distribution. P- and np-charts rely on the binomial distribution; whilst c- and u-charts rely on the Poisson distribution. The XmR chart makes no such assumption and instead uses the data themselves to provide empirical limits through calculation of an average moving range; whilst, for example, the p- and np-charts assume the variation to be a function of the location and plot theoretical limits that will not hold if the binomial assumption is violated. Technical details of the different types of control chart and the relevant assumptions can be widely found, *e.g.*[3,4].

In healthcare, more so than in the manufacturing birthplace of SPC, we will seldom be in a position to justify stringent assumptions, such as those of the binomial model, satisfactorily. The simplest control chart, the XmR chart also has the distinct advantage of having the least stringent assumptions attached to it. In fact the only assumption required is that a rational sampling and sub-grouping regime is used [11]. In this sense rational means taking into account the context for the data, sources of variation, and the questions to be addressed by the charts. Thus in the complex real world of healthcare, the robustness of the XmR chart to distribution of the data is invaluable. Furthermore, even if the assumptions of a specific model do hold, in most cases the XmR chart will yield identical results to the more restrictive chart [4]. With this in mind, for the rest of this article we will focus on the XmR chart.

For the XmR analysis of data with a natural ordering, it is important that global measures of dispersion, such as the overall standard deviation, are not used to in the calculation of control limits [12]. This is because such a global measure only makes sense in the context of an assumption that the data is homogeneous; whilst the primary question that the control chart is designed to answer is precisely this: is the data homogeneous, or are there signals within of heterogeneity – “special causes”. Instead the correct method for calculating the control limits for an XmR chart is via the average moving range [2,7,11]. This subtle distinction is of fundamental importance in the correct application of the methodology of SPC.

Whilst the XmR chart was originally formulated with time-series data in mind, its use has been advocated for data in which there is logical comparability but no inherent ordering of the data, provided the order in which the data is placed is not determined by the data themselves [4]. In this article we will explore quantitatively the consequences of the lack of natural ordering for the average moving range, both theoretically and via an example using real world data. We then discuss alternative approaches to the detection of special causes for data without a natural order.

## Methods

There are SPC charts for which permuting the order of the data does not affect the calculation of the control limits. These include the p-charts, np-charts, c-charts and u-charts, but as mentioned above, these charts rely on stringent assumptions that are unlikely to be met by real healthcare data. However, the control limits of the XmR chart are affected by permutations of the data, as is shown below.

Suppose that the data we are interested in, $\left\{x_{i},x_{2},\ldots ,x_{n}\right\}$ , do not possess a natural order. We may assume that the data are labelled in a non-descending order, i.e. that $x_{i}\leq x_{i+1}$. By choosing an ordering of the data, say *y*_*1*_*,y*_*2*_*,…,y*_*n*_; and then applying the method outlined in [4], we obtain a value for the average moving range $\bar{mR}$:

(1) $$\bar{mR}=\frac{1}{n-1}\sum_{j=1}^{n-1}\left|y_{j+1}-y_{j}\right|$$

There are at most *n*! distinct possible orderings of the data, giving at most $\frac{n!}{2}$ possible values for $\bar{mR}$ (the average moving range is invariant under a reversal of the order). There is, therefore, an order that results in the largest possible value of $\bar{mR}$, and an order that results in the smallest. It is of interest to calculate these extrema in order to understand the appropriateness of the average moving range as a measure of variation in the data.

The minimum $\bar{mR}$, ${\bar{mR}}_{\min}$, is easily seen to be $\frac{x_{n}-x_{1}}{n-1}$ - simply the difference between the smallest datum and the largest, averaged over the *n*-1 moving ranges. The maximum, ${\bar{mR}}_{\max}$ , is somewhat more difficult to establish. To describe the maximum we must introduce some notation. Let $\delta_{j}=\left|x_{j+1}-x_{j}\right|$ for *j* = 1,2,…,*n*-1. Also, let $s_{\max}=\left(n-1\right){\bar{mR}}_{\max}$ and $s_{\min}=\left(n-1\right){\bar{mR}}_{\min}$. The cases of *n* odd and even have to be considered separately.

**Case I:***n* is odd

It can be shown that, if $\delta_{\frac{n+1}{2}}\geq \delta_{\frac{n-1}{2}}$ then

(2) $$\begin{array}{c} S_{\max}=2\delta_{1}+4\delta_{2}+\ldots +\left(n-3\right)\delta_{\frac{n-3}{2}}+\left(n-2\right)\delta_{\frac{n-1}{2}}\\ +\left(n-1\right)\delta_{\frac{n+1}{2}}+\left(n-3\right)\delta_{\frac{n+3}{2}}+\ldots +4\delta_{n-2}+2\delta_{n-1} \end{array}$$

and if $\frac{\delta_{n+1}}{2}\leq \frac{\delta_{n-1}}{2}$, then

(3) $$\begin{array}{c} S_{\max}=2\delta_{1}+4\delta_{2}+\ldots +\left(n-3\right)\delta_{\frac{n-3}{2}}+\left(n-1\right)\delta_{\frac{n-1}{2}}\\ +\left(n-2\right)\delta_{\frac{n+1}{2}}+\left(n-3\right)\delta_{\frac{n+3}{2}}+\ldots +4\delta_{n-2}+2\delta_{n-1} \end{array}$$

Note that if the two central *δ* s are equal, these two expressions are the same.

**Case II:***n* is even

It can be shown that

(4) $$\begin{array}{c} S_{\max}=2\delta_{1}+4\delta_{2}+\ldots +\left(n-2\right)\delta_{\frac{n-2}{2}}+\left(n-1\right)\delta_{\frac{n}{2}}\\ +\left(n-2\right)\delta_{\frac{n+2}{2}}+\ldots +4\delta_{n-2}+2\delta_{n-1} \end{array}$$

Since $x_{n}-x_{1}=\delta_{1}+\delta_{2}+\ldots +\delta_{n-1}$ it is clear that the difference $S_{\max}-S_{\min}$ can be obtained by reducing all coefficients of the δ_j_ in the above expressions by one.

The distribution of the average moving ranges between the two extrema will necessarily depend on the underlying data, and the closed form distribution is not clear.

### Real world example

Taking a real world research example from a quality improvement initiative, running as part of the National Institute of Health Research (NIHR) Collaboration for Leadership in Applied Health Research and Care for Northwest London (CLAHRC NWL) we investigated the consequences of using an XmR chart analysis on data that possess no underlying order, but that are logically comparable. In an improvement initiative aiming to improve ward compliance with hospital trust policy, 23 wards over 4 hospital sites spread across northwest London entered compliance data on a weekly basis to a centralised, multi-user web platform tailored to meet the project requirements [13]. Note that for these data, there is no justification for the assumption that the probability of each patient’s care being compliant within a given ward is the same, and therefore we cannot use a p-chart in this case.

We calculate average overall compliance in each ward for a year period (01-04-2010 to 31-03-2011) to allow comparison of these multiple sites, and then investigate the effect of different orderings of these data items on the average moving range through a resampling without replacement (shuffling) algorithm.

## Results and discussion

The summary statistics for the set of ward percentage compliance figures over a year period are displayed in Table 1.

**Table 1:** Annual percentage compliance with hospital trust policy in 23 wards, rounded to 3 sf

	
Mean	33.7%
Median	27.9%
Standard Deviation	16.9%
Range	58.3%
Minimum	16.7%
Maximum	75%
Count	23

**Figure 1:**
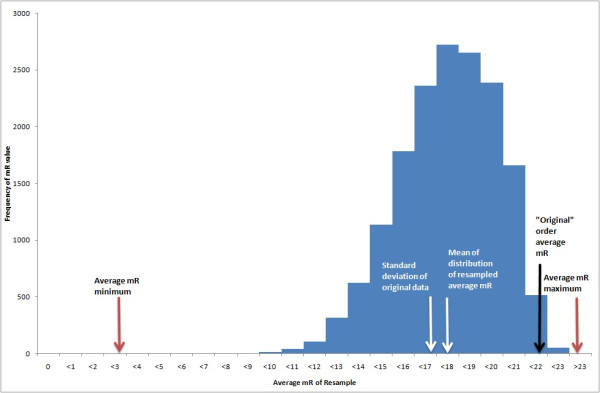
**A histogram of the distribution of**$\bar{mR}$**from a resampling without replacement exercise on a real world data set of the ward compliance with hospital trust policy.** This exemplifies the possible variation in the $\bar{mR}$ statistic that can arise when data are re-ordered, highlighting the extent of the problem when the data have no underlying order, such as time.

In order to apply an XmR chart analysis to this data set, the data must be placed in a specific order. The 23 wards can be ordered in $2.59\times 10^{22}$ (23!) possible ways. The order in which the wards were added into the web platform as they joined the initiative was used to provide an “original” ordering, with $\bar{mR}$ 21.7%. By resampling with replacement (shuffling) from the population of possible orders (in MS Excel using formulae) 16,383 sample orders were generated - this large data set representing a tiny fraction $\left(6.33\times 10^{-17}\%\right)$of all the possible configurations. It is important to note that within each ordering, all 23 data points are present precisely once each. The average moving range for each of these resamples and summary statistics of those average moving ranges are displayed as Table 2. In addition, a histogram of the shuffled $\bar{mR}$ values from these was plotted as Figure 1.

**Table 2:** Summary statistics for the average moving ranges from the resampling exercise, to 3sf except count

	
Mean	17.5%
Standard Error	0.0173%
Median	17.7%
Standard Deviation	2.21%
Range	16.1%
Minimum	6.70%
Maximum	22.8%
Count	16383

The formulae from the previous section show that the set of possible values of $\bar{mR}$ for this data has minimum 2.6% and maximum 23.1%, a range of 20.5%. This represents the degree of ambiguity in the calculation of the average moving range for this data set. The exact distribution of the $\bar{mR}$ values is not clear, and will depend on the underlying data. The lowest and highest values obtained in the distribution of the resamples were 6.7% and 22.8%, with the central 95% of sampled $\bar{mR}$ values falling between 12.8% and 21.2%. Therefore it is clear that the $\bar{mR}$ statistic is not robust to permuting the order of the data. It is now possible to examine the consequences of this fact for the control limits.

**Figure 2:**
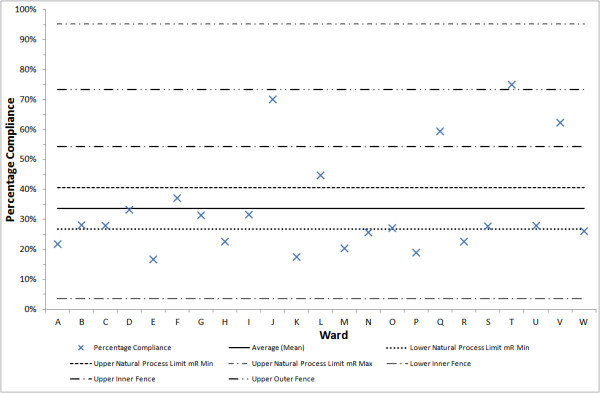
**An Average and SD chart of the dataset, with Tukey’s Fences superimposed; as well as control limits based on**${\bar{mR}}_{\min}$**and**${\bar{mR}}_{\max}$. This highlights the problem of using $\bar{mR}$ for datasets with no inherent order. Here the data are placed in “original” order. Limits falling outside of the range 0%-100% are not plotted. This shows the consequences of the issue highlighted in Figure 1, and suggests that one should instead fall back on classical outlier analyses in these instances.

Calculating the control limits using ${\bar{mR}}_{\min}$ yields 26.8% and 40.6% for the upper and lower limits respectively (width 13.8%). Similarly, calculating using ${\bar{mR}}_{\max}$ yields −27.7% and 95.2% (width 123%). These control limits can be seen in relation to the data in Figure 2 – note that the negative lower control limit is replaced by 0% in interpreting the chart in practice. As a consequence of simply permuting the order of the data, an absolute difference of 109 percent in the width of the control limits can be effected – 1.87 times the actual range of the data. Since the control limits are intended to convey information about the process that generated the data, it is clear that this level of ambiguity in the analysis renders it unacceptable as an approach to making decisions based on the data.

Since the XmR chart is not an appropriate way to analyse this data, how should one attempt to distinguish special causes from common causes in this case? Without a natural ordering, the problem becomes one of outlier detection, and an appropriate technique may be selected from the well developed literature on this issue [14]. Examples of the simplest outlier detection methods include: the 3-sigma rule derived from the properties of the normal distribution (or more generally using the Vysochanskii-Petunin inequality [15] for unimodally distributed data) – equivalently an average and standard deviation chart [11]; and Tukey’s method of “fences” [16]. Neither of these depend upon the ordering of the data. In this case, as the data is skewed, it is appropriate to apply Tukey’s method, in other words to define lower and upper “fences” at *Q*_*1*_*-k(Q*_*3*_*-Q*_*1*_*)* and *Q*_*3*_ *+ k(Q*_*3*_*-Q*_*1*_*)* respectively. Here *Q*_*1*_ and *Q*_*3*_ are the lower and upper quartiles of the data. Values of *k = 1.5* and *k = 3* are often taken to define “inner fences” and “outer fences”, these are plotted in Figure 2. Examining Figure 2 it is apparent that the number of points that should be investigated as potentially due to special causes using ${\bar{mR}}_{\min}$ XmR control limits, ${\bar{mR}}_{\max}$ XmR control limits, inner fences, outer fences are 14, 0, 4 and 1 respectively, out of 23 data points. This provides another means of understanding the lack of robustness of the XmR chart analysis to permutation of this data.

## Conclusions

In conclusion, usage of p-, np-, c- and u-charts for data without natural ordering proceeds precisely as for data endowed with a natural ordering such as time. This is not the case for the simplest but more distribution-robust control chart, the XmR chart. The control limits on an XmR chart are dependent on the ordering of the data, and this dependency is such that the ambiguity in “expected variation” (as quantified by the range of possible widths of the control limits) is large when working with data that have no inherent natural order. We have given a real data set for which this range is almost double the range of the actual data – clearly an unacceptable degree of ambiguity.

Thus when one is faced with a problem of distinguishing special from routine variation in a univariate data set with no time order, the individuals and moving range (XmR) chart is not appropriate, and simply using a random order that is not based on the magnitude of the values, as advocated in primer texts [4,11,12] is not sufficient to address this issue. In this case one should fall back on the usual outlier analyses available to the statistician, *e.g.*[14]. In the example data above we have applied the well known and simple method of “fences”, due to Tukey [16]. In practice, the choice of outlier detection methodology will depend on the particular application.

As such, for identification of potential special causes in a dataset we recommend that:

1) In time series data when there is limited or no knowledge of the distribution of the data, the XmR chart is the appropriate method of analysis, using the $\bar{mR}$ for construction of limits, thereby accounting for the underlying data order.

2) In data without a natural order, an appropriate outlier detection method should be selected instead of using XmR - some simple examples being a) Tukey’s method of “fences” b) the 3 sigma rule (note this corresponds to using an average and standard deviation chart). See [14] for further methods of outlier detection.

3) Authors should explicitly state the method used, including how control limits were calculated.

## Abbreviations

CLAHRC NWL, Collaboration for Leadership in Applied Health Research and Care for Northwest London; NIHR, National Institute for Health Research; $\bar{mR}$, Average moving range; SPC, Statistical process control; XmR, Individuals chart also known as X and moving range chart.

## Competing interests

The authors declare that they have no competing interests.

## Authors' contributions

All authors contributed to the design of the project. AP set up and implemented the resampling algorithms and wrote the first draft of the manuscript. TW determined the mathematical proofs in determination of the maxima for average moving range. All authors contributed to later drafts and gave final approval to the manuscript.

## Pre-publication history

The pre-publication history for this paper can be accessed here:

http://www.biomedcentral.com/1472-6947/12/86/prepub
